# Few-Electrode EEG from the Wearable Devices Using Domain Adaptation for Depression Detection

**DOI:** 10.3390/bios12121087

**Published:** 2022-11-28

**Authors:** Wei Wu, Longhua Ma, Bin Lian, Weiming Cai, Xianghong Zhao

**Affiliations:** 1 School of Information Science and Engineering, NingboTech University, Ningbo 315100, China; 2 School of Information Science and Engineering, Zhejiang Sci-Tech University, Hangzhou 310018, China

**Keywords:** domain adaptation, depression detection, few electrodes, electroencephalography

## Abstract

Nowadays, major depressive disorder (MDD) has become a crucial mental disease that endangers human health. Good results have been achieved by electroencephalogram (EEG) signals in the detection of depression. However, EEG signals are time-varying, and the distributions of the different subjects’ data are non-uniform, which poses a bad influence on depression detection. In this paper, the deep learning method with domain adaptation is applied to detect depression based on EEG signals. Firstly, the EEG signals are preprocessed and then transformed into pictures by two methods: the first one is to present the three channels of EEG separately in the same image, and the second one is the RGB synthesis of the three channels of EEG. Finally, the training and prediction are performed in the domain adaptation model. The results indicate that the domain adaptation model can effectively extract EEG features and obtain an average accuracy of 77.0 ± 9.7%. This paper proves that the domain adaptation method can effectively weaken the inherent differences of EEG signals, making the diagnosis of different users more accurate.

## 1. Introduction

Depression is a common mental illness that causes some degree of negative impact in various countries around the world. According to the World Health Organization, 300 million people worldwide already suffer from depression, and this number is increasing every year. Depression is present at all ages and harms generations [1]. People with depression sometimes cannot rationally control their words and actions, which can lead to very serious consequences. In most cases of suicide, it can be understood that the vast majority of suicides are caused by suffering from varying degrees of mental illness, of which depression is an important causative factor [2]. The main features of depression are prolonged depressed mood, slowed thinking, and cognitive impairment with negative attitudes towards many people and events [3]. This will definitely affect the life and work of the patient, which is detrimental to both the individual and society [4,5]. Depending on the severity of the depression, depression can be broadly classified as major depression, moderate depression, and mild depression [6]. In general, patients with major depression can be clearly detected by observing them over a period of time, but patients with moderate and mild depression are more difficult or hard to detect and can become more severe if they do not receive timely treatment [7]. In order to improve this situation, timely diagnosis and treatment are needed. The current mainstream diagnostic approach is based on the diagnostic and statistical manual of mental disorders (DSM-IV) [8] and other similar approaches to psychiatric rating scales [9]. Traditional testing methods are more subjective and less accurate, which does not allow for a timely diagnosis of the disease. Therefore, better solutions need to be proposed for the diagnosis of early depression.

Today’s methods of detecting depression rely on the experience of physicians and various psychiatric testing scales [10,11], and the diagnosed person may ignore the symptoms exhibited at an early stage and, thus, not be examined in a timely manner, while others may choose to avoid and not be willing to actively and truthfully speak about their condition because of fear psychology, which can largely affect the diagnostic results [12,13]. This calls for finding a method of judgment that is not altered by subjective consciousness. Many studies have shown that EEG signals play an adjunctive role in the detection and treatment of certain diseases.

EEG is a technique for non-invasively studying the electrophysiological dynamics of the brain and relating these dynamics to cognition and disease. EEG measures and records the electrical signals of the brain. When collecting EEG, the experimenter places small electrodes on the scalp. The electrodes attach to a machine that gives the doctor information about the brain’s activity [14]. Today there are a growing number of studies and applications of EEG that play an important role in personalized medicine or psychiatry, such as epilepsy, emotion classification, the diagnosis of sleep disorders, and the treatment of movement disorders [15,16,17]. However, the raw EEG signal usually contains various noises, which can affect the process and results of the study to some extent. Therefore, many researchers are undertaking efforts to achieve noise reduction. For example, for muscle interference, in addition to noise reduction by traditional methods, some studies are combining classical and effective methods, thus forming a new system, and are obtaining relatively better results in noise reduction [18,19]. Seok et al. summarize the development of motion artifact removal techniques to a certain extent, which has good results in practical applications. With the development of artificial intelligence, motion artifact removal techniques have good performance in the face of challenging problems [20]. Electrical source imaging (ESI) is also useful for noise removal from EEG signals. It is also increasing in the treatment of neurosciences such as epilepsy and in clinical applications [21,22].

EEG-based analysis and processing is also an approach to the detection and treatment of depression. Olbrich et al. proposed that EEG can be used to distinguish whether there is depression or not, and the test results are relatively good and can be standardized for identification [23]. Wu et al. proposed that coherence features extracted from EEG signals in the resting state are more reliable for the detection of major depression [24]. In recent years, with the development of the Internet of Things and sensors, it has become easier to identify depression by EEG, and Wei et al. designed a wearable device to collect EEG signals in real-time [25]. Cai et al. proposed a universal method for EEG-based depression detection, which proved to be effective in identifying depressed patients from a small number of conductively collected EEG signals [26]. In summary, it can be demonstrated that the EEG contains effective features for discriminating depression and that lightweight, non-invasive, low-cost wearable devices have some prospects for depression detection.

Depression detection based on EEG can be implemented with different algorithms and deep learning networks. When effective feature selection is performed on the EEG, the support vector machine (SVM), linear discriminant analysis (LDA), naive Bayes (NB), k-nearest neighbors (kNN), and decision tree (D3) can be used to make predictions with better results [27]. Akbari et al. suggested a novel strategy for the diagnosis of depression based on several geometric features derived from the EEG signal shape of the second-order differential plot (SODP) [28]. With the development of deep learning, many researchers are using deep network models for the detection of depression. Ay et al. used a deep hybrid model based on EEG using a combination of the convolutional neural network (CNN) and long-short-term memory (LSTM) for depression detection, which improved the accuracy [29]. Loh et al. used short-time Fourier transform (STFT) to process EEG and put the generated spectral images into a CNN model for training to improve the idea of implementing automatic depression detection [30]. However, there is not much attention or research on the adverse effects of EEG differences between users on depression detection. This will maybe hinder the application of EEG-based depression detection in real life.

Since EEG can be affected by individual differences such as age, gender, and life circumstances, it could lead to variability in EEG signals. Therefore, practical and effective treatments are needed to improve the accuracy of depression diagnosis within certain limits. To weaken the adverse effects of variability, transfer learning may be a good option [31]. Transfer learning can improve the performance of a model on a target domain by transferring knowledge contained in a different but related source domain, which reduces the dependence on the target domain [32]. In transfer learning, when the data distribution of the source and target are different, but the two tasks are the same, this particular type of transfer learning is called domain adaptation [33]. Domain adaptation has good results in solving the target domain with a large amount of unlabeled data and large differences from the source domain, and Ganin et al. introduced the application scenario of domain adaptation in the unsupervised case [34]. Some recent studies have shown that domain adaptation in deep networks can also learn features that can be transferred, which opens new avenues for deep network-based research [35]. However, a pressing problem of domain adaptation in deep networks is that the discrepancy between the source and target domains increases significantly as the number of layers deepens. Long et al. proposed a domain adaptation framework that could effectively reduce the discrepancy in deep networks by mapping the depth features of a specific layer to reproducing kernel Hilbert space (RKHSs), which can migrate the domain distribution among different features [36]. In conclusion, domain adaptation can be a good solution to the challenges posed by the variability of data distribution.

Wearable EEG, as a low-cost data signal, has great potential for application. However, the time-varying nature of each individual’s EEG signal and the non-uniform data distribution have a very negative impact on depression detection. After the EEG is captured by a simple, non-invasive wearable device for proper processing, it is then trained and predicted using a cross-user deep domain adaptation network based on the migration learning and kernel function embedding theory, which can effectively attenuate the variability of EEG signals from different users and enable more accurate diagnosis for different users. The main contributions of this paper are as follows:

- A convenient scheme for the online diagnosis of depression without restricting the user’s free movement
- Effective depression detection across users using domain adaptation methods.

## 2. Materials and Methods

### 2.1. Participants

**Figure 1:**
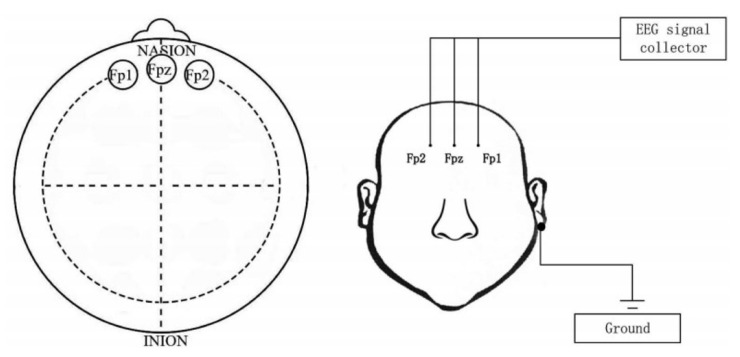
Brain acquisition location and placement of the three-electrode device. Reprinted with permission from Ref. [38]. Copyright 2019, copyright Shen et al.

In this experiment, the dataset used was the multi-model open dataset for mental-disorder analysis (MODMA) dataset from the Key Laboratory of Wearable Equipment in the Gansu Province, and the EEG signals collected at a resting state with three electrodes were the main choice [37]. The dataset consists of 29 healthy subjects and 26 depressed subjects (19 males and 10 females of 29 healthy subjects and 15 males and 11 females of 26 depressed subjects). Table 1 shows the demographic characteristics of the participants. EEG signals were acquired mainly from three parts of the prefrontal lobe of the brain (Fp1, Fpz, and Fp2), and the approximate location of the brain acquisition and the placement of the acquisition device are shown in Figure 1. During the acquisition process, data were collected from participants in the resting state in order to exclude, to some extent, the influence of non-EEG factors.

### 2.2. Data Preprocessing

Since the collected EEG samples are divided into healthy samples and depression samples, and the data are stored in text files, and then in the process of domain adaptation, the convolution-based neural network is mainly used, so it is necessary to convert the text type data into image type; such data types can be trained and predicted in adaptive networks. The following describes two methods of processing data into pictures.

#### 2.2.1. Three Channel Data Merge Chart

**Figure 2:**
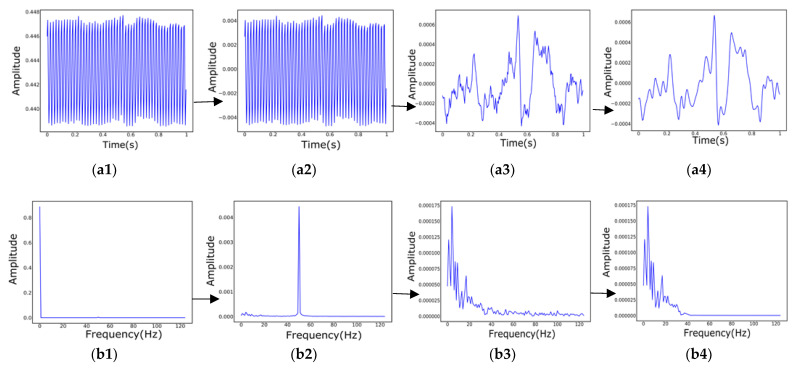
The filtering time and frequency diagram of the preprocessing process (the above shows the filtering process of the Fpz electrode EEG of sample data (subject ID: 02010003), and other samples that are similar). (**a1**–**a4**) Time domain filtering process; (**a1**) Original time domain diagram; (**a2**) After 1 Hz high-pass filtering; (**a3**) After [48, 52] trap filtering; (**a4**) After [0.5, 35] bandpass filtering; (**b1**–**b4**) The four graphs are, respectively, the frequency domain waveforms of the time domain graphs (**a1**–**a4**).

**Figure 3:**
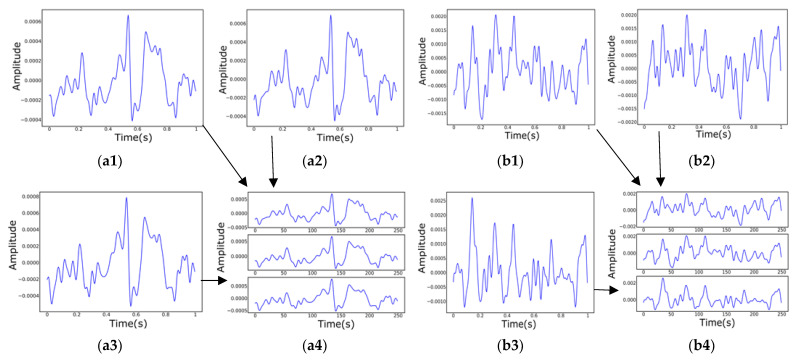
Draw Fp1, Fpz, and Fp2 on the same picture in the sequence. (**a1**–**a4**) The three channels of data for depression sample (subject ID: 02010003) are plotted separately; (**a1**) Fpz; (**a2**) Fp1; (**a3**) Fp2; (**a4**) Three diagramming; (**b1**–**b4**) The three channels of data for health sample (subject ID: 02020004) are plotted separately.

The data of the three channels are drawn separately in a single picture, each with a data interval of one second. Since the range of values of the original data is different, the original data needs to be normalized to the same range, and the set range is [0, 1]. Additionally, during the data collection process, the subjects do not show healthy or depressed EEG emotions more accurately at the beginning or the end of the collection, taking into account the influence of external factors. This is somewhat misleading in determining whether depression is present, so the first and last parts of the collected sample data are removed. The intermediate data obtained in this way will be somewhat representative. This experiment focuses on taking out the data starting at position 30% to position 70% of each sample. Since the sampling frequency of the data acquisition is 250 Hz, 250 data points are selected to draw a picture of the one-second EEG signal. Then, the EEG signal is filtered, mainly to filter out features that are not relevant to the prediction results. The original signal is first high pass filtered with a cutoff frequency of 1 Hz, then trap filtered, and its frequency range is set to [48, 52] Hz. Finally, the band-pass is filtered with a frequency range set to [0.5, 35] Hz. Figure 2 mainly shows the time-frequency diagram of the filtering process, and finally, as in Figure 3, three columns of data are drawn in the same picture. Finally, the domain adaptation model is trained on black-and-white images. The main purpose is to prevent the model from learning unnecessary color features, which will make the detection results more convincing.

#### 2.2.2. Synthesis by RGB

**Figure 4:**
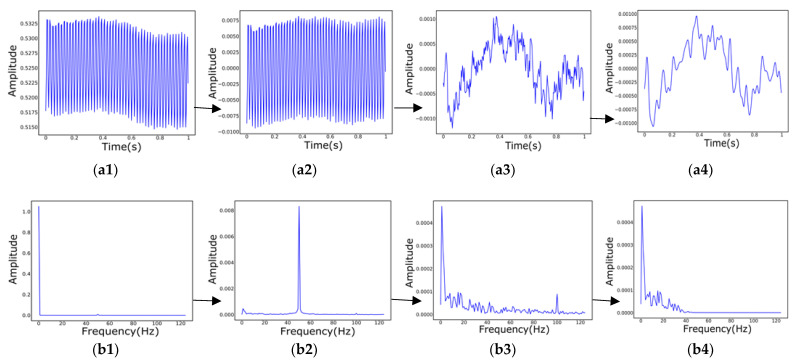
The filtering time and frequency diagram of the preprocessing process (the above shows the filtering process of the Fpz electrode EEG of sample data (subject ID: 02010005). In the time domain filtering process, axes are added to each filter map when displayed. However, the axes are not needed for RGB synthesis to exclude the influence of other factors of the synthesis map on the experimental results). (**a1**–**a4**) Time domain filtering process; (**b1**–**b4**) The four graphs are, respectively, the frequency domain waveforms of the time domain graphs (**a1**–**a4**).

**Figure 5:**
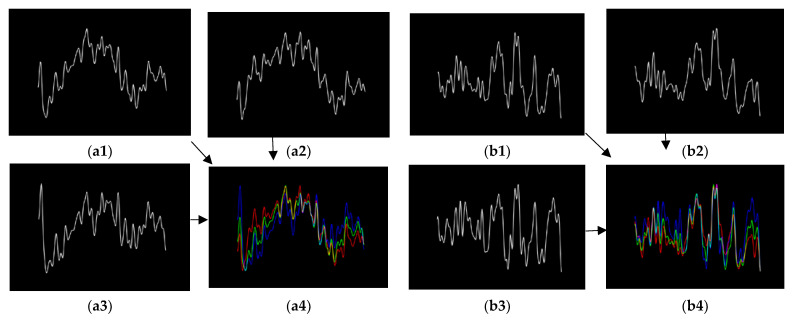
Combination of Fp1, Fpz, and Fp2 into RGB images (the background color is set to black in order to better show the color change in the RGB synthesis. Additionally, these graphs have no axes). (**a1**–**a4**) The three channels of data for depression sample (subject ID: 02010005) are plotted separately; (**a1**) Fpz; (**a2**) Fp1; (**a3**) Fp2; (**a4**) RGB synthesis; (**b1**–**b4**) and data synthesis by RGB for three channels of health sample (subject ID: 02020007).

The most important difference between this data processing method and the previous one is that the Fp1, Fpz, and Fp2 of the EEG signal, respectively, corresponding to the three channels of RGB to synthesize the picture. The other methods of processing the original text data at the beginning are the same, but when plotting, the coordinate system of the time-domain filtering process is removed to facilitate the final RGB synthesis and to avoid the influence of the coordinate system on the experimental results. Figure 4 mainly shows the respective distributions of the time domain and frequency domain of the data before and after filtering. When the picture is synthesized, the same will also turn the filtered data drawn out picture into a black and white picture. The main thing is to set the gray value to 0 at the position with the waveform and the gray value of the other backgrounds to 255 to obtain a single-layer picture. Finally, Figure 5 shows the use of OpenCV to synthesize a single-layer image drawn from the filtered data of three channels into an image by RGB.

### 2.3. Domain Adaptation

In domain adaptation, since the distribution of the data in the source and target domains is different, it is important to find ways to reduce this gap so that the model can easily perform a specific task on the data in the target domain. The data in the source and target domains are projected into the common space, which in turn, makes the difference between the data in the source and the target domains of this space smaller. This can be treated as a similar dataset that can be trained with various classifiers in this space. This provides an effective option for performing related tasks on data in unknown domains.

This experiment mainly adopts the domain adaptation model proposed in the literature, which is the deep adaptation network (DAN) [39]. The DAN is designed based on the classical AlexNet [40]. AlexNet contains eight neural network layers, mainly consisting of five convolutional layers in the front and three fully connected layers in the back. Generally, the front layers of a deep neural network are used to extract the basic features of the data and play the same role in other tasks, so the parameters that have been learned can be left unchanged. Additionally, some of the middle layers learn features that may be more consistent with specific tasks, and fine-tuning can be used to allow these layers to learn some data features for new tasks as well. In order to achieve better transfer learning, the last few fully connected layers are mainly used to change the parameters by the designed loss function. These may also be some of the ideas of the DAN design.

Compared with the traditional convolutional neural network, DAN can obtain more deep features of the data, which can extend the deep convolutional network to the adaptive network and improve the portability of the neural network to a certain extent. In neural networks, the extracted features will proceed from shallow to deep as the levels continue to deepen, but at the same time, it also brings the variability of levels to become larger and larger, leading to a significant decrease in the portability of the network. Adaptive networks map data from different domains to a new domain so that they can be trained and predicted in different domain data, and similarly, the key to the robustness of the adaptive network is to reduce the difference. In DAN, a new multiple kernel variant of the maximum mean discrepancies (MK-MMD) is proposed to reduce the discrepancy between the source and target domains, which in turn, effectively improves the accuracy of the network prediction. Multiple kernels can greatly improve the adaptive efficiency compared to a single kernel and will have more than the expected effects for processing more complex data. In this paper, DAN will be applied to EEG analysis, and experimental comparison results with other domain adaptation models will be presented in the experimental phase.

**Figure 6:**
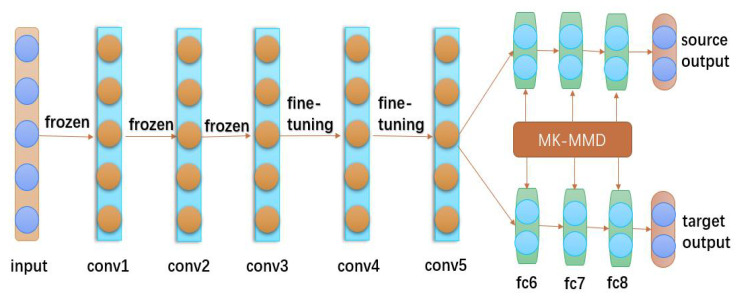
Structure of the DAN model with transferable features.

The structure of DAN Is mainly divided into three parts, as shown in Figure 6. The first part is composed of three convolutional layers conv1-conv3. The extracted features are the basic features of the data, so these layers are frozen. After other data passes through this part, the extracted features are also general. The second part is composed of two convolutional layers conv4-conv5. The features extracted in this part have a certain depth, so these layers are fine-tuned to change the relevant parameters, but these features are slightly less transferable. The third part consists of three fully connected layers fc6-fc8, which are designed for specific experiments, but these layers are not transferable, so these layers are parameterized by MK-MMD [39], and MK-MMD can enhance the transferability of the feature representation in deep neural networks. The squared formulation of MK-MMD is defined as

(1) $$d_{k}^{2}\left(p,q\right)≜∥E_{p}\left[\phi \left(x^{s}\right)]-E_{q}[\phi \left(x^{t}\right)\right]{∥}_{{ℋ}_{k}}^{2}$$

where ${ℋ}_{k}$ denotes the reproducing kernel Hilbert space (RKHS) endowed with a characteristic kernel *k*. The mean embedding of the distribution p in ${ℋ}_{k}$ is a unique element $\;\mu_{k}\left(p\right)$ such that $E_{x∼p}f\left(x\right)=\langle f\left(x\right),\mu_{k}\left(p\right)\rangle_{{ℋ}_{k}}$ for all $f\in {ℋ}_{k}.$ The MK-MMD $d_{k}\left(p,q\right)$ between the probability distributions p and q is defined as the RKHS distance between the mean embeddings of p and q. Another key point about this formula is that p=q iff $d_{k}^{2}\left(p,q\right)=0$. The characteristic kernel associated with the feature map $\phi ,k\left(x^{s},x^{t}\right)=\langle \phi \left(x^{s}\right),\phi \left(x^{t}\right)\rangle$ is defined as the convex combination of m positive semi-definite (PSD) kernels $\left\{k_{u}\right\}$,

(2) $$\kappa ≜\left\{k=\overset{m}{\underset{u=1}{\sum }}\beta_{u}k_{u}:\overset{m}{\underset{u=1}{\sum }}\beta_{u}=1,\beta_{u}\geq 0,\forall u\right\}$$

where the constraints on coefficients $\left\{\beta_{u}\right\}$ are imposed to guarantee that the derived multi-kernel k is characteristic. The multi-kernel k can be used to enhance MK-MMD performance with different cores.

### 2.4. Experiments

This section focuses on the use of three domain adaptation models, DAN [39], DANN (domain adversarial neural network) [34], and DeepCoral (correlation alignment for deep domain adaptation) [41], for the processed EEG data for training and prediction. Relevant basic data processing, sample selection and assignment, and comparison settings were performed during the experiments, mainly considering the effects of these settings on the experimental results, excluding unnecessary external factors, and analyzing the obtained experimental results.

#### 2.4.1. Experimental Setup

When the processed data are trained and tested by the domain adaptation model, some parameters of the neural network need to be set. To enable the network to be fully trained, the number of network cycles is set to 500, which allows for the iterations of the parameters of each layer to increasingly match the relevant characteristics of the training data. The base network for setting up the domain adaptation model is resnet34, which is a simpler network compared to resnet50 to avoid overfitting. To fully reflect the various features of the training data in the neural network during training, this requires setting the batch size to be relatively small for each training. In order to make full use of the GPU training model to a certain extent, the num_works is set to three, which can effectively accelerate the training process. In order to reduce the difference between the source and target domains in the adaptive network, the three domain adaptation models have their own different loss functions, among which the DAN model chooses the loss function MK-MMD, which enables the third part of the model to better transfer learning in different data distributions.

#### 2.4.2. Data Distribution

The data were divided into the source and target domains, which both contain two classifications of health and depression, and 16 samples were selected from all the sample images, with 8 healthy and 8 depressed samples. The first allocation is that the sample data are set in a 7:1 ratio of training to testing. The training set includes 7 healthy samples and 7 depressed samples, and the remaining healthy sample and one depressed sample are set as the test set. The second allocation is that the sample data is set in a 4:4 ratio of training to testing, which means that the healthy and depressed samples are divided equally.

## 3. Results

By training two kinds of image data with adaptive networks and then testing them on unfamiliar images, it can be observed that the two methods of processing data have different effects on prediction accuracy, and different data allocation ratios also have some effects on the experimental results.

**Figure 7:**
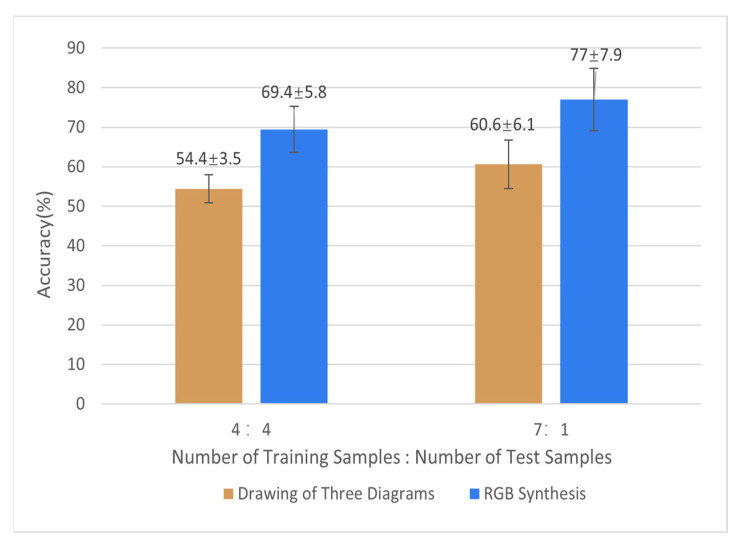
Prediction accuracy of three-map plotting and RGB synthesis under different sample ratios. (Assuming that each group contains a healthy sample and a depression sample, 4:4 means that four groups are the training set and the other four groups are the test set; 7:1 means that the seven groups are the training set and the other one group is the test set. Additionally, the healthy and depressed samples in all groups were from different participants).

**Figure 8:**
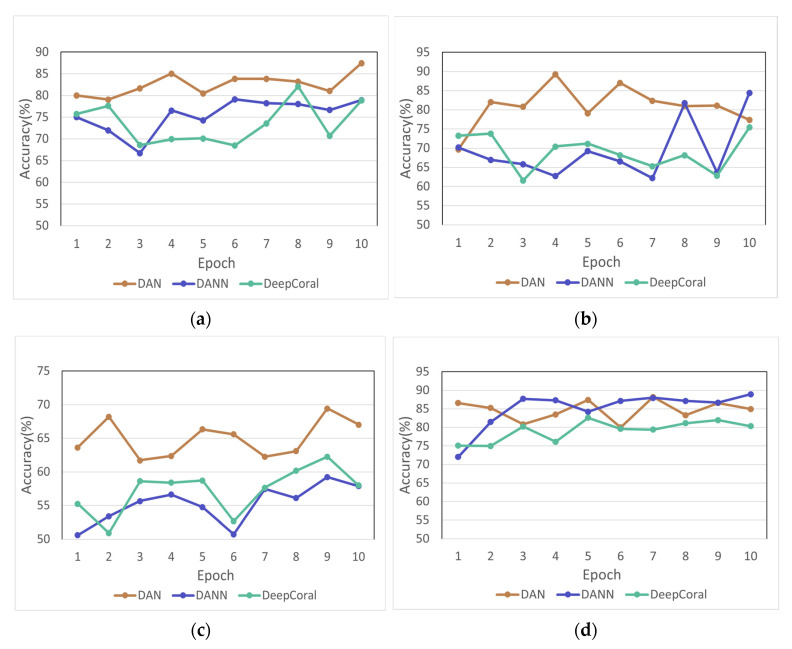
Variation in prediction accuracy with the number of training sessions in four groups. (**a**) Group 1; (**b**) Group 2; (**c**) Group 3; (**d**) Group 4.

In Figure 7, it can be seen that the accuracy of drawing three columns of data in the same picture is lower than that of the RGB synthesis, which is also in line with general cognition. RGB synthesis is more in line with the relevant characteristics of computer vision. The RGB synthesized image data were trained and predicted in the three models, and the relationship between the number of training and the prediction accuracy was obtained, as shown in Figure 8. In Figure 8, it can be found that the prediction accuracy of the DAN model is higher compared to DANN and DeepCoral, which indicates that the DAN model is more suitable to deal with the domain adaptation problem of the RGB synthesized images. During the experiments, it was found that different training and testing samples were selected from the total samples after passing through the adaptive network to obtain different accuracy rates. The prediction accuracy values of some different sample combinations are listed in Table 2, where S and T represent the source and target domains, respectively, and the numbers after them indicate that they are randomly from different combinations. Additionally, each grouping includes 16 samples, of which eight are healthy, and eight are the depressed samples, followed by seven healthy samples and seven depressed samples in S, and one healthy sample and one depressed sample in T. After a series of experiments, it can be found that the prediction accuracy of DAN is generally higher among the three adaptive models, so it can be ascertained that the DAN model can better extract the features of different data distributions, and the prediction of unknown data is more accurate. Additionally, it can be found in Figure 8 that the accuracy distributions of subplots (a,b,d) and subplot © are different. The reason for this may be the wide variation in the EEG patterns of each individual. The EEG signals of the patients with different degrees of depression are somewhat specific, and even a very small number of patients have a part in EEG characteristics similar to those of healthy users. The mechanism of depression is not clear at present, and the EEG itself is a non-stationary signal. The differences in the manifestation of depressive symptoms among patients are relatively large. This paper focuses on how to improve the accuracy of depression detection among different users. Using this method can serve to improve the accuracy rate of most users. This can also prove the meaning of the experiment and the validity and potential of the method in another way.

## 4. Discussion and Conclusions

Depression is a major health problem for millions of people, and this number is getting bigger, and there is a certain impact on the development and progress of society, so it must be diagnosed and treated in time. Additionally, early depression is more easily cured so that the damage of depression can be kept within a certain range and avoid bigger tragedies. The current mainstream method of depression diagnosis is manually intensive, and the result depends mainly on the doctor’s experience, which has some drawbacks. In daily life, an easy, low-cost, wearable depression detection device that can detect depression in time and allow depression to be detected at a curable stage is essential. Additionally, the detection of depression based on EEG signals is generally more reliable. Many studies have also shown that EEG signals contain some features of emotional states and mental disorders. However, the actual EEG acquisition process can mix in some noise, so a series of processing and analysis of the raw signal is needed before the neural network can be used to learn the features of depression. It may be more convincing to obtain the results this way.

Applying the domain adaptation approach to EEG signal-based depression diagnosis can better attenuate the effect of EEG signal variability on diagnostic accuracy. Such a model trained on limited known data can show better diagnostic results in undetected individuals, giving a scientific and objective approach to depression detection, which may be valuable for the development of medical devices and personal detection devices for depression. After a series of experiments, the proposed method can effectively weaken the variability of the EEG signals of different users and diagnose other users more accurately. However, during the experiments, the data set used is relatively small, which will affect the generalizability of the detection system to some extent. Additionally, the model is based on a deep learning algorithm, whose data computation complexity may be high and requires a relatively long time to learn the data features. In the future, more sample EEG datasets will be collected and produced, and a wider range of participants will be made available. Models trained with such datasets may have significantly improved accuracy when faced with predictions from different categories of people. We will continue to try and optimize the model in order to better achieve the online deployment of rapid diagnosis and to allow easy mobile devices to make timely and accurate predictions locally.

## Figures and Tables

**Table 1:** Participant demographic characteristics.

Characteristics	Depression Group	Health Group
Number (male:female)	15:11	19:10
Age	16–56	19–51
Number of years of education	6–19	12–19

**Table 2:** Prediction accuracy of different sample combinations under the three models. (**a**) First six groups’ prediction accuracy; (**b**) The prediction accuracy of the last five groups; and the average accuracy of all subgroups.

Model	S1→T1	S2→T2	S3→T3	S4→T4	S5→T5	S6→T6
DAN	87.4%	69.6%	89.9%	89.3%	78.7%	68.8%
DAN	79.1%	64.9%	81.4%	84.4%	61.9%	64.6%
DeepCoral	82.1%	67.3%	82.8%	75.4%	72.4%	62.2%
(**a**)						
**Model**	**S7→T7**	**S8→T8**	**S9→T9**	**S10→T10**	**S11→T11**	**Average**
DAN	69.4%	62.1%	75.7%	68.0%	88.2%	77.0%
DAN	59.2%	63.7%	65.7%	61.9%	88.9%	70.5%
DeepCoral	62.3%	50.8%	65.2%	57.0%	82.6%	69.1%
(**b**)						

## Data Availability

Publicly available datasets were analyzed in this study. This data can be found here: http://modma.lzu.edu.cn/data/index/ (accessed on 25 November 2022).
